# Respiratory muscle weakness and its association with exercise capacity in patients with chronic obstructive pulmonary disease

**DOI:** 10.1111/crj.13449

**Published:** 2021-10-11

**Authors:** Yves de Souza, Maria Eduarda Suzana, Stefany Medeiros, Joseane Macedo, Cláudia Henrique da Costa

**Affiliations:** ^1^ Physiotherapy Department Veiga de Almeida University Rio de Janeiro Brazil; ^2^ Pulmonary Medicine Department State University of Rio de Janeiro Brazil

**Keywords:** COPD, exercise capacity, muscle weakness, respiratory exercise, respiratory muscles

## Abstract

**Introduction:**

Although COPD patients commonly present respiratory complaints despite pharmacological treatment, dyspnea does not correlate directly and linearly with spirometric data, a fact that makes it difficult to select patients for pulmonary rehabilitation. Thus, seems logical that the measurement of respiratory muscle strength could help in this initial assessment if it presents a good correlation with exercise capacity. The aim of this study is to assess whether patients with muscle weakness, characterized as a reduction in maximal inspiratory pressure (PImax) below 70% of predicted value, have a good relationship between the assessed respiratory muscle strength and the exercise capacity measured by the 6‐min walk test (6MWT) in patients with COPD.

**Methods:**

Patients diagnosed with COPD according to the 2019 *Global Initiative for Chronic Obstructive Lung Disease* (GOLD) on regular use of their medications, without exacerbations for 3 months or more and with respiratory muscle weakness (PImax < 70% of predicted) performed 6MWT in a 30‐m‐long flat corridor.

**Results:**

Data from 81 patients were analyzed. There was a strong correlation between the distance of the 6MWD with the PImax (*r* = 0.764, *p* < 0.0001). When separating the sample by the 350‐m cut in the 6MWD, we found that the patients with the worst performance in the test are those who present the greatest respiratory muscle weakness.

**Conclusion:**

PImax correlates well with exercise capacity, and patients with respiratory muscle weakness could be referred to a pulmonary rehabilitation protocol tied to inspiratory muscle training.

## 1 INTRODUCTION

Dyspnea is the main limiting symptom of exercise capacity in COPD, leading to a chronic intolerance to physical activities.^1^ However, respiratory symptoms are not directly related to forced expiratory volume in first second (FEV1), which makes it difficult to classify patients based on data collected on spirometry.^2^ Currently, the Global Strategy for Prevention, Diagnosis and Management of COPD (GOLD) suggests that pharmacological treatment should be performed according to a flowchart that assesses the persistence or not of dyspnea or exacerbation despite the use of bronchodilators associated or not with inhaled corticosteroids. Thus, the search for an objective marker of exercise intolerance that correlates with dyspnea and exercise capacity, and that is easy to apply, can help in the programming of respiratory rehabilitation, indicating when it is necessary to introduce muscle strengthening exercises. The aim of this study is to evaluate the relationship between respiratory muscle weakness, clinically characterized as a reduction in maximal inspiratory pressure (PImax) below 70% of predicted value,^3^ and exercise capacity in patients with COPD.^4^, ^5^

## 2 METHODS

This is an observational study with a cross‐sectional analytical design. The sample consisted of patients consulted at the outpatient clinic between February and December 2019, who agreed to participate in the study. A free and informed consent form, informing the ethical and legal aspects of the research, was signed by all participants. The project was approved by the Ethics Committee on Research in Human Beings of the Institution under number 050.991/2016.

Inclusion criteria were as follows: Patients aged 40–80 years diagnosed with COPD according to the 2019 *Global Initiative for Chronic Obstructive Lung Disease* (GOLD)^6^; on regular use of their medications, without exacerbations for 3 months or more; respiratory muscle weakness (PImax < 70% of predicted)^7^; and without osteomyoarticular and neurofunctional alterations that would compromise the performance of the evaluation tests. Patients with diagnoses of psychiatric or cognitive diseases that compromised the understanding of the instructions for evaluations and interventions, progressive neurological diseases, neuromuscular diseases, vestibular disorders, and orthopedic conditions that compromised gait were excluded.

Patients who met the inclusion criteria were evaluated on a single occasion. The method of evaluation of respiratory muscle strength was through the measurement of PImax, measured according to the guidelines of the European Respiratory *Society* (ERS),^8^ using a specific mouthpiece. The test was repeated three times with rest time between inspirations of less than 2 min; the highest value was selected as the PImax and also expressed as a percentage of the predicted value.^7^ Exercise capacity was evaluated using the 6‐min walk test (6MWT) distance in a 30‐m‐long flat corridor, and patients were instructed according to the American *Thoracic Society* ATS/ERS guidelines,^9^ using Brazilian reference values.^10^

Regarding the sample size, a calculation performed (http://www.sample-size.net/correlation-sample-size/) using alpha of 0.01 and beta of 0.10, aiming to achieve a correlation >0.50 between exercise capacity and respiratory muscle strength indicated the need for a sample of 52 patients.

For statistical analysis, the Shapiro–Wilk test was used to analyze the normality of the distribution of the data, with description of the data in average and standard or median deviation and interquartile interval. Considering that the literature has a value called “poor distance” of the 6MWD, which has as a cutting line the highest mortality in patients with COPD 6MWD < 350 m,^11^ the patients were categorized into two groups according to the 6MWD: individuals with 6MWD above and below 350 m. Comparison between these two groups was performed by the unpaired Student's *t* test or the Mann–Whitney test for continuous variables. The correlations were evaluated by Pearson's coefficient. Statistical analysis was performed using the GraphPad Prism 6.0 statistical package (GraphPad 6.0, Software, Inc., USA). Statistical significance was determined as *p* < 0.05.

## 3 RESULTS

We included 81 patients in the study, diagnosed with respiratory muscle weakness, presenting PImax < 70% of the predicted value. In general, the sample presented BMI above the normal range, and no significant difference between the groups divided by the 6MWD, except for the values of the distance itself and the PImax (Table 1).

**TABLE 1:** Baseline characteristics of patients

Variables	6MWD > 350 m (*n* = 55)	6MWD < 350 m (*n* = 26)	*p*‐value
Gender (M/F)	29/26	16/10	
Age (years)	67 ± 6	67 ± 7	0.6457
BMI (kg/m^2^)	29 ± 4	30 ± 6	0.3454
FEV1 (%predicted)	48 ± 19	50 ± 18	0.2771
FVC (%predicted)	86 ± 15	84 ± 13	0.3548
FEV1/FVC (%)	55 ± 12	59 ± 13	0.1154
Hand grip strength (Kg/F)	25 ± 7	26 ± 6	0.7325
Hand grip strength (%predicted)	76 ± 16	78 ± 13	0.2574
6MWD (m)	411 ± 47	286 ± 49	<0.0001
6MWD (%predicted)	70 ± 14	42 ± 12	<0.0001
PImax (cmH_2_O)	60 ± 7	39 ± 6	<0.0001
PImax (%predicted)	57 ± 8	36 ± 5	<0.0001

*Note*: Data are presented as mean ± SD.

Abbreviations: BMI, body mass index; FEV1, forced expiratory volume in 1st second; FVC, forced vital capacity; 6MWD, 6‐min walking distance; PImax, maximal inspiratory mouth pressure; %predicted, percentage of the predicted normal value.

**FIGURE 1:**
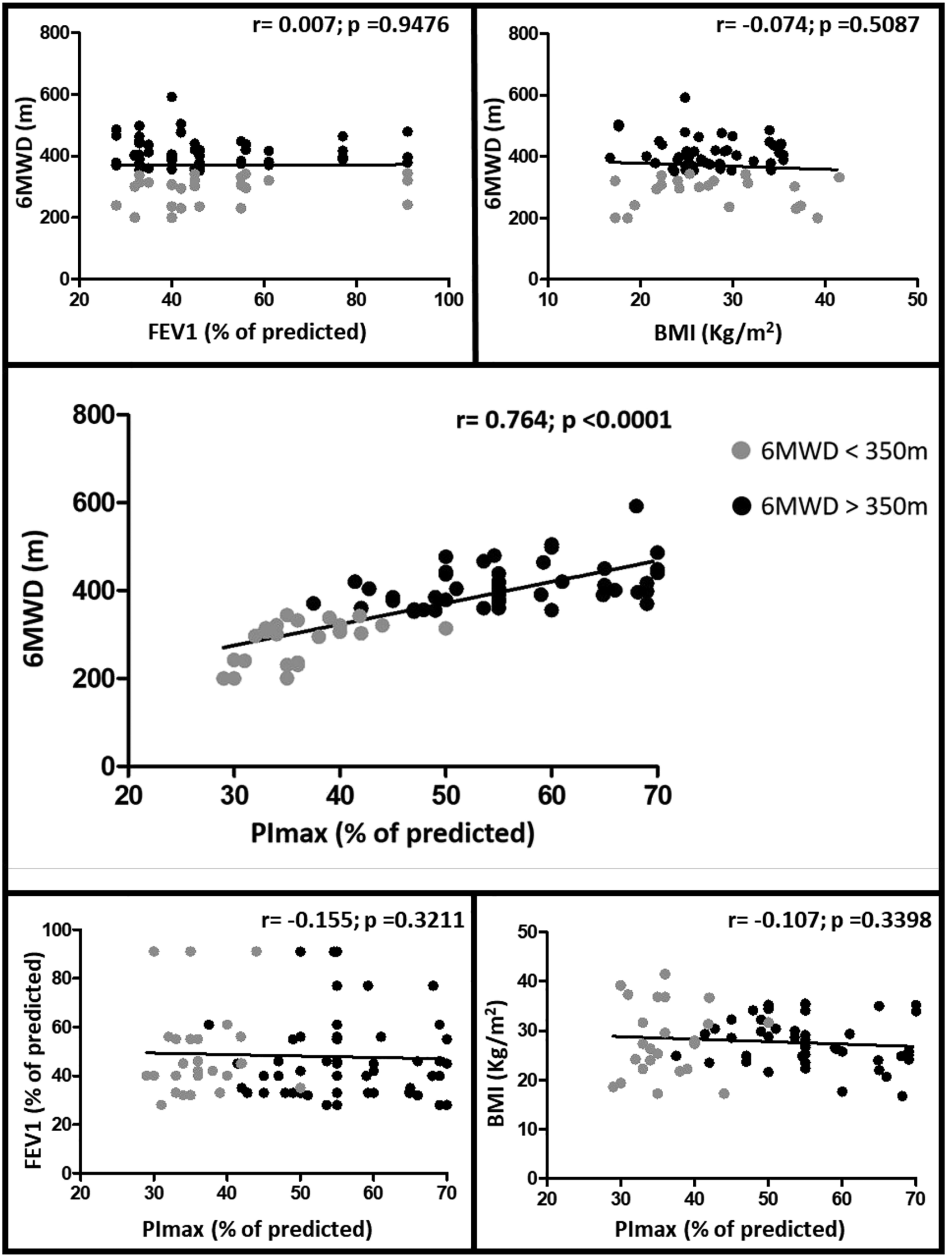
Correlations between 6MWD expressed in meters and PImax expressed as a % of predicted value. 6MWD, 6‐min walking distance; PImax, maximal inspiratory mouth pressure. It was observed a good correlation between Pimax and 6MWT, but there is no correlation between other variables

The group of patients with 6MWD < 350 m presented significantly lower values of PImax, with a strong correlation *r* = 0.764; *p* < 0.0001 (Figure 1). We did not find a significant correlation between PImax and VEF1 (*r* = −0.155; *p* = 0.3211) and BMI (*r* = −0.107; *p* = 0.3398) or with any other variables studied.

## 4 DISCUSSION

The association between respiratory muscle weakness and low exercise capacity in patients with chronic obstructive pulmonary disease (COPD) was analyzed transversely in 81 patients who had PImax < 70% of the predicted value. There was a strong correlation between the distance of the 6‐min walk test (6MWD) with the PImax (*r* = 0.764). When separating the sample by the 350‐m cut in the 6MWD, we found that the patients with the worst performance in the test are those who present the greatest respiratory muscle weakness. These data are in accordance with a study that found a value of 350 m as a inflection point below which the risk of death and hospitalizations increases almost linearly as the walking distance decreases.^11^ We reinforce the importance of assessing respiratory muscle strength in patients with COPD. Interestingly, there was no reduction in peripheral muscle strength, measured by hand grip, in patients who walked less than 350 m in the walk test.

Recently, it has been demonstrated that clinical interventions that increase 6MWD and PImax are determining factors for improving exercise tolerance in patients with COPD.^12^ In addition, new studies are published each year demonstrating that, through inspiratory muscle training (IMT), a specific training modality of respiratory muscles, an improvement in exercise capacity is achieved through increased strength and mobility of respiratory muscles, generating consequent decrease in the sensation of dyspnea.^13^

Charususin et al. have also correlated these variables, pointing to dyspnea as the main limiting factor during the performance of exercises in patients with COPD, having as a direct cause inspiratory muscle weakness.^14^ After a pulmonary rehabilitation protocol tied to IMT, patients presented increased MIP, lower scores for dyspnea and, consequently, better performance in the proposed exercises.^15^ When the intervention is isolated IMT, gains in exercise capacity are also found.^16^

Our study has some limitations, such as the fact that it was carried out in a single center, that it included only patients with muscle weakness, and that it did not carry out other assessments such as the measurement of lean body mass. However, the results were quite expressive and deserve to be evaluated in future studies.

## 5 CONCLUSION

In conclusion, there is a good correlation between respiratory muscle strength and 6MWD, and patients low PImax could be referred to a pulmonary rehabilitation protocol tied to inspiratory muscle training.

## CONFLICT OF INTEREST

The authors declared no conflict of interest.

## AUTHOR CONTRIBUTIONS

Yves de Souza conceived of the presented idea. Maria Eduarda Suzana, Stefany Medeiros and Joseane Macedo performed the data collection. Yves de Souza, Maria Eduarda Suzana and Claudia Henrique da Costa verified the analytical methods. Claudia Henrique da Costa supervised the project. All authors discussed the results and contributed to the final manuscript.

## ETHICS STATEMENT

The project was approved by the Ethics Committee on Research in Human Beings of Pedro Ernesto University Hospital under number 050.991/2016.

## TRIAL REGISTRATION

RBR‐10nyzcqc

## Data Availability

Data sharing is not applicable to this article as no datasets were generated or analyzed during the current study.
